# Opinion Mining From Social Media Short Texts: Does Collective Intelligence Beat Deep Learning?

**DOI:** 10.3389/frobt.2018.00138

**Published:** 2019-01-22

**Authors:** Nicolas Tsapatsoulis, Constantinos Djouvas

**Affiliations:** Image Retrieval and Collective Intelligence Lab, Department of Communication and Internet Studies, Cyprus University of Technology, Limassol, Cyprus

**Keywords:** opinion mining, social media messages, sentiment analysis, collective intelligence, deep learning, crowdsourcing

## Abstract

The era of big data has, among others, three characteristics: the huge amounts of data created every day and in every form by everyday people, artificial intelligence tools to mine information from those data and effective algorithms that allow this data mining in real or close to real time. On the other hand, opinion mining in social media is nowadays an important parameter of social media marketing. Digital media giants such as Google and Facebook developed and employed their own tools for that purpose. These tools are based on publicly available software libraries and tools such as *Word2Vec* (or *Doc2Vec*) and *fasttext*, which emphasize topic modeling and extract low-level features using deep learning approaches. So far, researchers have focused their efforts on opinion mining and especially on sentiment analysis of tweets. This trend reflects the availability of the Twitter API that simplifies automatic data (tweet) collection and testing of the proposed algorithms in real situations. However, if we are really interested in realistic opinion mining we should consider mining opinions from social media platforms such as Facebook and Instagram, which are far more popular among everyday people. The basic purpose of this paper is to compare various kinds of low-level features, including those extracted through deep learning, as in *fasttext* and *Doc2Vec*, and keywords suggested by the crowd, called *crowd lexicon* herein, through a crowdsourcing platform. The application target is sentiment analysis of tweets and Facebook comments on commercial products. We also compare several machine learning methods for the creation of sentiment analysis models and conclude that, even in the era of big data, allowing people to annotate (a small portion of) data would allow effective artificial intelligence tools to be developed using the learning by example paradigm.

## 1. Introduction

Big Data does not only refer to dealing with enormous data sets in terms of data capturing, data storage, and data processing (De Mauro et al., 2015; Hashem et al., 2015) but it is also strongly related with predictive analytics (Yaqoob et al., 2016) and data mining (Fan and Bifet, 2013). Artificial intelligence tools, on the one hand, are strongly related with data mining and artificial intelligence is nowadays ranked first among the top-10 technology (Buzzwords, 2019). A great portion of the huge amounts of online data that led us to the era of Big Data is created within or with the aid of social media platforms. Among those data we see customer reviews, comments and opinions about products, people, and events. All this information, if properly processed, is invaluable for businesses, governments, and individuals. As a result, opinion mining in social media became one of the primary pillars of social media marketing (Zafarani et al., 2014). It is not a surprise that digital media giants such as Google and Facebook developed and employed their own artificial intelligence tools for that purpose. Going one step further they created and made publicly available software libraries and tools such as *Word2Vec* (Mikolov et al., 2013) or *Doc2Vec* (Le and Mikolov, 2014) and *fasttext* (Joulin et al., 2017) to show that they are at the front of applied research and to increase their prestige among the academic community. The aforementioned tools basically emphasize topic modeling through word embeddings; the latter being low-level feature representations of digital words extracted with the aid of deep learning approaches (Socher et al., 2013). Nevertheless, there is an increasing tendency nowadays to develop intelligent data mining applications by combining data crawled from social media sites with crowdtagging (Giannoulakis and Tsapatsoulis, 2016a,b; Ntalianis and Tsapatsoulis, 2016).

In one of our previous studies (Tsapatsoulis and Djouvas, 2017), we have shown that tokens identified through crowd-tagging of tweets can be used as an index of terms for training effective tweet classification models in a learning by example paradigm. We had concluded that this type of indices, i.e., human-indicated terms, are probably the best feature set one can use for tweet classification. We empirically proved this through extended experimentation, in which we compared the human-created index of terms with many different automatically extracted feature sets in a machine learning scenario using three different classifiers. In this work we extend that study by investigating a more difficult problem: that of sentiment classification of tweets into three challenging categories (anger, hate, neutral). We also examine the problem of sentiment classification of Facebook comments regarding commercial products. The human-created indices are developed by using crowd-tagging through a well-known dedicated crowdsourcing platform to allow full reproduction of the hereby suggested empirical study. However, the real purpose of this study is to empirically compare the power of collective intelligence, as expressed by the crowd-collected keywords from tweets and Facebook comments, with that of deep learning, as expressed through the modeling of those short texts (i.e, tweets and Facebook comments) with character n-grams as in *Doc2Vec* and the fastText (2018) classifier. To the best of our knowledge none of the three research actions briefly mentioned above have been reported before in the corresponding literature.

## 2. Theoretical Framework and Related Work

This paper investigates the importance of collective knowledge in creating artificial intelligence systems that operate in a “Big Data” environment. In this context it is imperative to review practical tools that allow collective knowledge - intelligence to be gathered. Crowdsourcing platforms are the contemporary solution of this demand. The second pillar of the literature review, presented next, focuses on short text classification methods, and techniques. Since the majority of these methods were applied to tweets, it is obvious that our emphasis is also given there. Methods that combine crowdsourcing and tweet classification are also examined extensively.

### 2.1. Crowdsourcing and Crowdtagging

The theoretical roots of crowdsourcing are located in the so-called “wisdom of crowds” theory which was first introduced by Surowiecki (2005). The term “crowdsourcing” itself, a composite word consisting of the words “crowd” and “outsourcing,” was coined by two editors at *Wired*, namely Jeff Howe and Mark Robinson, to define the process through which businesses were using the Internet to “outsource work to the crowd”. In particular, Jeff Howe (2008) defines crowdsourcing as: “*…the act of a company or institution taking a function once performed by employees and outsourcing it to an undefined (and generally large) network of people in the form of an open call. …The crucial prerequisite is the use of the open call format and the large network of potential laborers.”* Nowadays a variety of crowdsourcing platforms are available in the Web (Doan et al., 2011) to allow crowdsourcing to take place in a few steps: Amazon Mechanical Turk (MTurk, n.d.), TurKit (n.d.), uTest (n.d.) and Figure-eight (n.d.) are a few of them.

Kleemann et al. (2008) were among the first that explored the phenomenon of crowdsourcing for research and marketing purposes. In their article they sought a more precise definition of crowdsourcing, cataloged some of its forms, and differentiated them from satellite phenomena. They concluded with a discussion regarding potential consequences, negative and positive, of a wider use of crowdsourcing, especially in marketing. Brabham (2009) investigated public involvement for urban planning as a means to collect intellect from a population in ways that person-to-person planning meetings fail. He suggested that crowdsourcing provides a distributed problem-solving paradigm for business that enables seeking creative solutions for public planning projects through citizens' active involvement in that process. As a proof of concept he analyzed crowdsourcing in a hypothetical neighborhood planning scenario. He concluded his work with a discussion on the challenges that effective crowdsourcing implementation (at that time) posed. Vukovic (2009) reviewed a variety of crowdsourcing platforms through a crowdsourcing scenario for requirements' collection aiming at the development of generic crowdsourcing services in the cloud. He identified a set of critical features that crowdsourcing platforms should have and he evaluated these platforms against those features. He concluded with a taxonomy proposal for the crowdsourcing platforms while he outlined research challenges for enhancing crowdsourcing capabilities.

The Amazon Mechanical Turk (MTurk) was probably the first dedicated crowdsourcing platform that was used in research experimentation and especially for data annotation (crowdtagging). Hsueh et al. (2009) compared the quality of annotation data from expert and non-expert annotators, recruited through MTurk, in the context of classifying sentiments from political blog snippets. Noise level in the data, sentiment ambiguity and lexical uncertainty were identified as the three main factors that impede harnessing high-quality annotations from the crowd (non-experts). Finin et al. (2010) used both Amazon Mechanical Turk and Figure-eight (previously named CrowdFlower) to gather named entity annotations of Twitter status updates taking into account the informal and abbreviated nature of named entities in tweets. According to the authors, the collected annotations and the proposed annotation approaches provide a first step toward the full study of named entity recognition in social media like Facebook and Twitter that takes advantage of the crowd intelligence.

Crowdsourcing in marketing, based on tweets, was examined by Machedon et al. (2013), aiming at the development of techniques and tools for automatic topic and sentiment identification in social media communications using supervised machine-learning classifiers. The authors concluded that effective classifiers can be created using the crowdsourced training data. Although this work presents some similarities with our work, we should note here that our emphasis is put on the comparison of keyword selection for lexicon-based classifiers with classifiers that use low-level features extracted through deep learning. Furthermore, we also examine classification of Facebook comments and, as we will see later, these comments generally differ from tweets.

Borromeo and Toyama (2014) used crowdsourcing to account for the huge effort that is required for manual sentiment analysis of written texts. They claim that the performance of automatic systems for this task is poor except for systems that are based on the learning by example paradigm. They compared the results obtained by crowdsourcing, manual sentiment analysis and an automatic sentiment analysis system and they concluded that both paid and volunteer-based crowdsourced sentiment analysis is significantly more effective than automatic sentiment analysis but cannot achieve high accuracy with respect to manual annotation by experts.

Crowd-tagging was mostly applied in the specific task of image annotation. For the completeness of our literature review we report here some recent work, but many other reports do exist. Mitry et al. (2016) compared the accuracy of crowdsourced image classification with that of experts. They used 100 retinal fundus photograph images selected by two experts. Each annotator was asked to classify 84 retinal images while the ability of annotators to correctly classify those images was first evaluated on 16 practice—training images. The study concluded that the performance of naive individuals to retinal image classifications was comparable to that of experts. Giuffrida et al. (2018) measured the inconsistency among experienced and non-experienced users in a leaf-counting task of images of Arabidopsis Thaliana. According to their results, everyday people can provide accurate leaf counts. Maier-Hein et al. (2014) investigated the effectiveness of large-scale crowdsourcing on labeling endoscopic images and concluded that non-trained workers perform comparably to medical experts. In a survey by Cabrall et al. (2018) on categorizing driving scenarios, they used the crowd to annotate features from driving scenes such as the presence of other road users and bicycles, pedestrians etc. They used the Crowdflower platform (now *Figure-eight*) to categorize large amounts of videos with diverse driving scene contents. As usual, the Gold Test Questions in Crowdflower were used to verify that the annotators perform well in their job. The results indicated that crowdsourcing through Crowdflower was effective in categorizing naturalistic driving scene contents.

### 2.2. Short Text Classification

In Table 1 we show the six categories of features that can be used for text classification according to Layton et al. (2011). Those belonging to the categories of structure, content and semantics are intended for large texts while the ones belonging to the categories of word, character and syntax are well-suited for short texts. In our previous work (Tsapatsoulis and Djouvas, 2017) we performed an evaluation of the features in these three categories in order to identify the ones that are more suitable for the task of sentiment classification of tweets into three broad classes (positive, negative, and neutral). We concluded that numerical features, that is frequencies and ratios, do not carry any discriminative power while methods based on indices of words (unigrams—tokens) or *n*-grams of characters perform much better. We also found that bigram-based indices (proposed to account for negation) give only minor accuracy improvements compared to unigram indices in contrast to what Pak and Paroubek (2010) suggested.

**Table 1:** Frequently used features for text analysis as categorized by Tsapatsoulis and Djouvas (2017).

**Word level**	**Character level**	**Syntax**
Mean word length	Character n-grams	Frequency of function words
Number of hapax Legomena (n.d.)	Ratio of alphabetic characters	Frequency of punctuation marks
Number of hapax dislegomena	Ratio of character repetition	Part of speech (POS) tags
Ratio of distinct words	Ratio of digit characters	Ratio of spelling errors
Ratio of short words	Ratio of emoticons	Total number of lines
Skip grams	Ratio of special characters	Total number of sentences
Total number of unique words	Ratio of tab space characters	
Total number of words	Ratio of upper case letters	
Word distribution per length	Ratio of white space characters	
Word frequencies	Total number of characters	
Word *n*-grams	Vowel combinations	
**Structure**	**Content**	**Semantics**
Characters per paragraph	Number of abbreviations	Hyperonyms of words
Number of quoted content	Number of age based words	Hyponyms of words
Number of paragraphs	Number of gender based words	Synonyms of words
Number of sentences	Number of keywords	
Sentences per paragraph	Number of slang words	
Words per paragraph	Number of stopwords	

In general, we can identify three different approaches for short text classification. Lexicon-based methods are more common in sentiment analysis. The lexicons consist of emotionally-colored terms showing sentiment polarity. These terms are usually suggested by human experts and are used in a rule-based manner or through learned classifier models to assess the sentiment of short texts such as tweets. The lexicon-based approach is rather effective for classifying short texts into two different classes, e.g., positive and negative sentiment, but as new categories are included the classification performance drops steeply. Machine learning approaches use pairs of texts and relate labels - tags to train classification models. The key in these methods is the features that are extracted from the texts to train/feed the classifier. Given that the proposed method belongs to this type of approach, our literature review emphasizes the feature sets used in these methods. Finally, social network approaches are targeting social media messages and involve techniques from the field of Social Network Analysis (SNA) to extract social content and characteristics. Social network properties are not sufficient for short text classification and, therefore, social network approaches are combined with methods from the other two categories. Nevertheless, not a solid improvement has been reported when social network characteristics are combined with lexicon-based or machine learning approaches.

Mac Kim and Calvo (2010) evaluated various automatic sentiment polarity detection methods applied on students' responses to unit of study evaluations (USE). They started from the five universal emotion categories (Karpouzis et al., 2000)—anger, fear, joy, sadness and surprise—and they further classified joy and surprise as related to positive polarity while anger, fear and sadness were classified in the negative polarity. The performance of the developed category-based and dimension-based emotion prediction models was assessed on the 2940 textual responses of the students. The WordNet-Affect was utilized as a linguistic lexical resource for the category-based modeling while two dimensionality reduction techniques, namely latent semantic analysis (LSA) and non-negative matrix factorization (NMF), were also applied in the dimension-based modeling. In the latter case, the Affective Norm for English Words (ANEW) normative database, composed of affective terms, was used. Both the NMF-based categorical modeling and the dimensional modeling resulted in performances above the chance level (50% in that case).

Barbosa and Feng (2010) used pairs of text snippets and noisy labels, obtained from three websites, to develop a sentiment classification model. An additional set of 2000 manually labeled tweets were equally split and utilized for model-tuning on tweets and for testing. Their feature set consists primarily of Part of Speech (POS) tags of tweet words along with other syntax analysis features. They have also used network activity characteristics like retweets, hashtags, links, punctuation and exclamation marks as well as prior polarity of words found in sentiment lexicons. Agarwal et al. (2011) compared the POS-based method of Barbosa and Feng (2010) with the unigram baseline on both tree kernel and feature-based machine learning models. They showed that the models developed with POS tags and syntax characteristics outperformed the unigram-based ones. Their feature analysis revealed that the most discriminative features are those that combine the prior polarity of words and their parts-of-speech tags. They also concluded that sentiment classification of tweets is similar to sentiment classification of other text types. The authors, however, did extend preprocessing on the tweets, such as emoticons, acronyms and slang word translation to formal language words and, thus, they alleviated the majority of Twitter data's distinct characteristics. In other words, they actually transformed the problem of sentiment analysis of tweets into a typical text-classification task. Finally, we should remind here that, as in all POS-based approaches, language dependency makes the specific method non-generalizable to languages other than English.

Narayanan et al. (2013) experimented with a Naive Bayes classifier for sentiment analysis of movie reviews, aiming to find the most suitable feature set-data preprocessing combination. They found that effective negation handling along with word n-grams and feature selection through mutual information metrics, results in a clear improvement in sentiment prediction accuracy which reached 88.80% on the IMDB movie reviews dataset. Thus, they concluded that a highly effective and efficient sentiment classifier can be built using a simple Naive Bayes model, which has linear training and testing time complexities. According to the authors, their method can be generalized to several different text classification problems whenever a combination of speed, simplicity and accuracy is required. Compared with the work of Tsapatsoulis and Djouvas (2017) this work shows that accounting for negation and using some kind of syntactic analysis could be helpful in other types of short texts, in contrast to what happens with tweets.

Stavrianou et al. (2014) initially experimented with natural language processing (NLP) methods for sentiment analysis of tweets but they soon recognized, soon, that NLP techniques alone do not provide the desired accuracy of sentiment prediction on unseen cases. In order to account for this ineffectiveness, they proposed a hybrid method in which the results of natural language analysis, mainly POS-related features, feed a machine learning classifier. Although they observed a slight improvement on the sentiment classification performance, the most important finding was that NLP features obtained through syntactic analysis do not fit well with machine learning classifiers in the learning by example paradigm. Thus, it would be better to keep NLP as a data preprocessing stage rather than as a dedicated feature extractor. Shirbhate and Deshmukh (2016) also tried to incorporate NLP into their system. They trained a Naive Bayes classifier to categorize positive, negative and neutral tweets referring to customer opinions regarding cameras, mobiles and movies. Their feature set consists of unigrams and POS tags. They also applied filtering based on mutual information measures for feature selection. A prediction accuracy of 89% on a dataset consisting of 2, 000 tweets was reported. However, the small test set composed from 250 tweets, which leads to a proportion of training:test set equal to 88:12, along with the experimentation with a single classifier, i.e., Naive Bayes, limits the validity of their conclusions.

Hamdan et al. (2015) experimented extensively on a variety of low-level feature categories for a two-class sentiment classification of tweets. They used an adapted logistic regression classifier fed with combined word *n*-grams, lexicons, Z score and semantic features, such as topic features and the distribution of tweet tokens into the Brown collection (Brown et al., 1992). They have also taken into account negation during data preprocessing. They found that the lexicon-based approach, i.e., the use of sentiment lexicons as an index of terms, provided the best results. Their work is, indeed, informative, well-developed and related to the current study in terms of its aims. However, in the real world, the neutral tweets and comments constitute a high percentage of short-texts/messages exchanged every day in social media platforms. Thus, a two-class tweet classification in which the neutral category is excluded would definitely lead to misleading conclusions and, more importantly, it would be difficult to apply in realistic problems related to sentiment analysis of short messages.

Prusa et al. (2015) approached the problem of tweet classification from a different perspective: They denoted that due to the variability of tweets' contents, word- or character-based indices generate thousands of features while each tweet instance, due to the character length limit, contained only a few features of the entire feature set. Thus, the feature vector representation of each tweet will be sparse. Starting from this observation, they explored the influence of filter-based feature selection techniques on the tweet classification performance, using ten feature subsets of varying lengths and four different machine learning methods. They empirically showed that feature selection significantly improves classification performance and they concluded that both the selection of ranker and the feature subset length affect tweet classification. Deep learning methods for feature extraction do, in fact, carry out the above-mentioned procedure implicitly but in a much more systematic way. Thus, a comparison with methods that are based on feature extraction through deep learning, as we do in the current study, covers fully the method of Prusa et al. (2015).

## 3. Methodology

The basic assumption in this work is that collective intelligence regarding the appropriate sentiments of short texts, including tweets and Facebook comments, can be obtained with the aid of crowd-tagging within a dedicated crowdsourcing platform. In addition, we argue that the tokens used by humans for the classification of those short texts can be used to represent them as multidimensional points in high informative vector spaces (Salton et al., 1975). This representation allows for effective models (classifiers) to be learned in the learning by example scenario. Our main hypothesis is that these classifiers surpass, in terms of effectiveness, classifiers learned with low-level features of the short texts, as in *Doc2Vec*, even in the case where they are combined with deep learning architectures which have embedded and especially designed classifiers, as in *fasttext*. The methodology we follow to confirm or reject this hypothesis consists of four stages and is described below.

### 3.1. Mathematical Formulation

Let us assume a set of *N* short texts (i.e, facebook comments, tweets, etc.) 𝔻 = {*d*_1_, *d*_2_, …, *d*_*N*_} and a corresponding set of labels 𝕃 = {*l*_1_, *l*_2_, …, *l*_*N*_} so that every short text *d*_*i*_ is assigned a label *l*_*i*_ from a limited set ℂ = {*c*_1_, *c*_2_, …, *c*_*K*_}, corresponding to short texts' sentiments.

Let us also denote with 𝔽 = {*f*_1_, *f*_2_, …, *f*_*M*_} a set of transforming (or feature extraction) functions $f_{j}\left(𝔻\right)=\left\{{\vec{x}}_{1}^{j},{\vec{x}}_{2}^{j},\ldots ,{\vec{x}}_{N}^{j}\right\},j=1,2,\ldots ,M$ so that every short text *d*_*i*_ is represented as a vector ${\vec{x}}_{i}^{j}$ in a vector space.

The purpose of the current study is to identify the function *f*_*opt*_(𝔻) which maximizes short text classification in terms of accuracy of label prediction for a given classifier *T*, that is:

(1) $$\begin{array}{l} f_{opt}\left(𝔻\right)=\underset{j}{\mathrm{argmax}}\{\frac{1}{N}\overset{N}{\underset{i=1}{\sum }}O\left(f_{j}\left(d_{i}\right)\right)\} \end{array}$$

where

(2) $$o\left(f_{i}\left(d_{i}\right)\right)=\{\begin{array}{cc} 1, & if\;T\left(f_{i}\left(d_{i}\right)\right)=l_{i}\\ 0 & otherwise \end{array}$$

and $T\left(f_{j}\left(d_{i}\right)\right)=T\left({\vec{x}}_{i}^{j}\right)={\hat{l}}_{i},\;{\hat{l}}_{i}\in \mathbb{C}$.

### 3.2. Data Collection and Crowdtagging

In this study we used two datasets crawled from Twitter and Facebook and annotated with the aid of the *Figure-eight* (previously known as *Crowdflower*) crowdsourcing platform. The main characteristics of these datasets are shown in Tables 2, 3.

**Table 2:** Dataset #1: Facebook comments on commercial products.

	**Sentiment**					**Total**
	**Comparative**	**Ironic**	**Negative**	**Neutral**	**Positive**	
Gold	613	111	929	1,509	1,351	4,513
Crowd	362	50	1,443	1,162	1,496	4,513
Agreed	282	11	865	920	1,173	3,251
Recall	0.4600	0.0991	0.9311	0.6097	0.8682	0.7203
Precision	0.7790	0.2200	0.5994	0.7917	0.7841	0.7203
Training set	153	–	572	613	765	2,103
Test set	129	–	293	307	408	1,137

*Comparison with “gold standard” (students' assessment) is also shown*.

**Table 3:** Dataset #2: Tweets.

	**Sentiment**							**Total**
	**Anger**	**Disgust**	**Fear**	**Hate**	**Neutral**	**Sarcasm**	**Other**	
Fully agreed	202	27	3	128	335	43	539	1,277
Partially agreed	495	95	23	245	456	170	547	2,031
Contradicting								692
PRR	0.2898	0.2213	0.1154	0.3431	0.4235	0.2019		
Training set	429	–	–	247	502	–		1,178
Test set	268	–	–	126	289	–		683

*The degree of agreement between the annotators is also shown*.

The first dataset consists of Facebook comments about commercial electronic comments of a well-known multinational company. The comments were manually collected and stored in *.csv* files by students of the Cyprus University of Technology in the framework of the course “CIS 459: Natural Language Processing” during the period from October 2017 to January 2018. A subset of 4,513 comments was assessed by the students regarding their sentiment category as indicated in Table 2. We denote this initial dataset evaluation in terms of the expressed sentiment as our “gold standard.”

The second dataset consists of 4,000 tweets selected from an original dataset of 32 million tweets acquired through the Twitter streaming API during the period of March 2017 to April 2017 in the framework of the ENCASE (2016) project (see also Founta et al., 2018). The first step of the selection processes was to filter out some undoubted spam entries by discarding tweets with more than four hashtags, tweets with lengths of < 80 characters and native retweets (i.e., tweets that contain the “retweeted_status” field).

In addition to spam filtering, two more filtering criteria were applied in order to account for the fact that the great majority of the 32 million tweets were neutral in terms of sentiment. Toward this end, tweets underwent a preprocessing step, during which each tweet was augmented with two arguments: (a) polarity, and (b) number of inappropriate words (counter). The former was calculated using the TextBlob (n.d.) Python library. *TextBlob* produces a polarity output in the range of [−1.0, 1.0]. The latter parameters were created using two dictionaries containing Hate base (n.d.) and offensive words (Denn et al., 2015). All words in a tweet were stemmed and lower cased and matched against the two dictionaries; matching entries were counted and the final score was added to the augmented tweet. Using the two injected variables, tweets in this dataset are filtered so that they have a polarity in the range [−1, −0.7] and at least 1 offensive word. No filtration or processing on the users was applied; thus, more than one tweet from the same user might appear in the dataset.

**Figure 1:**
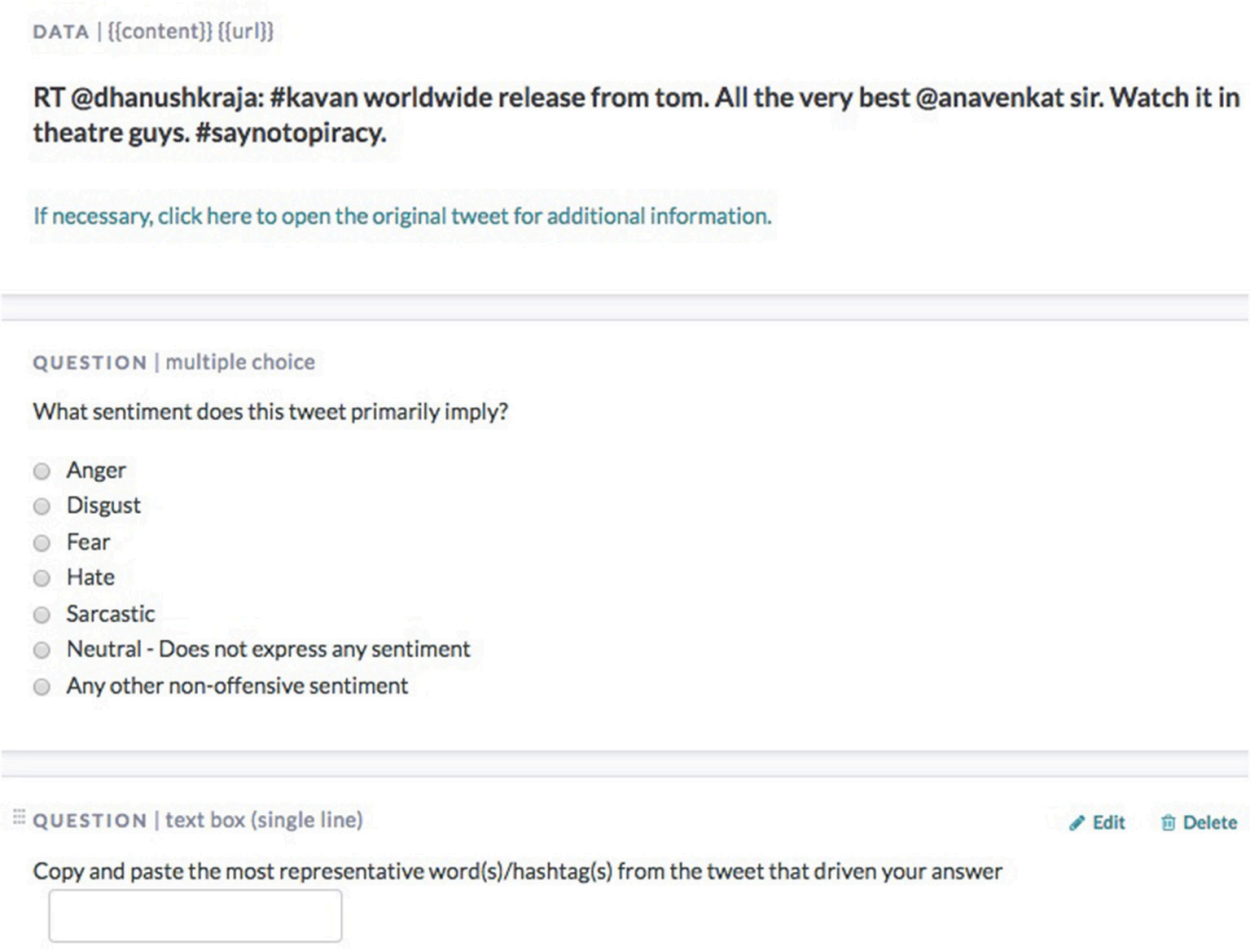
A snapshot of the crowd-tagging process that took place through *Figure-eight*.

Both datasets were uploaded to *Figure-eight* for sentiment labeling and crowdtagging as indicated in Figure 1. This figure shows the tweets' project but a similar design was also adopted for the Facebook comments. Every shot text was assessed from at least three annotators. The number of annotators does not really affect the identified sentiment category but it does increase the total number of (different) tokens that annotators used in crowdtagging. This, in turn, allows for different token selection strategies to form the *crowd lexicon*. Thus, a token can be added to the crowd lexicon in case it is suggested by all annotators during crowdtagging (full agreement), the majority of annotators or at least two annotators (Ntalianis et al., 2014).

As we see in Tables 2, 3, sentiment labeling proved to be a difficult task even for humans. Regarding the Facebook comments dataset, agreement of the majority of annotators (indicated as “Crowd” in Table 2) with the gold standard (“Gold”) was achieved on 3,251 comments (72.03%). However, the level of agreement varies significantly across the various sentiments. Identifying “Irony” in FB comments seems to be impossible, justifying that irony is not one of the universally recognizable sentiments (Cowie et al., 2001) even in written texts. The comparative comments present also low recall value (0.46) but the precision score is quite high (0.779). Some of the neutral comments are understood by the annotators as negative; this results in low recall value (0.6097) for the neutral comments and low precision value (0.5994) for the negative comments. Thus, we used the agreed comments, excluding the “irony” ones, to train and test our classifiers as explained in section 3.4.

The case of tweets was somehow different. For this dataset we did not have a gold standard, thus we decided to keep only those tweets for which the majority of annotators (crowd) agreed on the expressed sentiment. Through this process, 17.6% of the tweets were removed as being contradictory regarding their sentiments (see Table 3 for the absolute numbers). We also removed the tweets categorized as “Other” since their sentiment could not recognized and, obviously could not form a concrete and well defined category on its own. Given that there was no gold standard we decided to define a pseudo-recall rate (PRR) as follows:

(3) $$\begin{array}{l} PRR\left[s\right]=\frac{N_{F}\left[s\right]}{N_{P}\left[s\right]+N_{F}\left[s\right]} \end{array}$$

where *PRR*[*s*] denotes the pseudo-recall rate for sentiment *s* and *N*_*F*_[*s*] and *N*_*P*_[*s*] are, respectively, the numbers of tweets that were fully and partially agreed upon by the crowd that they express sentiment *s*.

As can be seen in Table 3 the *PRR* for all sentiments was rather low and, in some cases, as for the sentiments “Fear” and “Sarcastic,” was extremely low. This indicates the difficulty of classifying tweets into real sentiment categories and not into broad ones, such as “Positive” and “Negative,” as was done in the majority of the previous studies. Another important conclusion we can draw from Table 3 is that the “Fear” sentiment, although it is considered one of the universal sentiments (Cowie et al., 2001), is neither easily expressed nor easily identified in short written messages. On the contrary contrary, “Hate” is more easily understood in small written texts, although is not of one of the universal sentiments. This finding is in agreement with a recent study by Founta et al. (2018).

Since the absolute number of agreed tweets (*N*_*F*_) and the *PRR* of sentiments “Fear,” “Disgust,” and “Sarcasm” were both too low, we decided to exclude these sentiments for the experimentation. Thus, we ended up with a total number of 1,861 tweets, which were used for training and testing our models (see further details in section 3.4).

### 3.3. Vector Space Models and Word Embeddings

In our previous study (Tsapatsoulis and Djouvas, 2017), we showed that, among a variety of low-level features, the ones that are best suited for tweet classification are the unigrams that are either indicated by humans, denoted as *Human Index* (HI) or *Crowd Lexicon* (CL), or composed of the *Globally Most Frequent Tokens* (GMFT) in the training set. These two types of features, along with the classic *Bag of Words* (BoW) method that makes use of all tokens and their respective TF-IDF values (Maas et al., 2011), are compared with *fasttext* and *Doc2Vec* that are based on character *n*-gram features extracted through deep learning. Since GMFT, CL and BoW feature sets are all based on tokens, the corresponding trained classification schemes are, in fact, Vector Space Model representations. On the other hand, both *fasttext* and *Doc2Vec* are based on the Word Embeddings scheme.

The GMFT, CL, and BoW tokens of tweets and Facebook comments are utilized to construct indices of terms {*t*_1_, *t*_2_, …, *t*_*Q*_} in order to represent those short texts as high-dimensional points in a vector space model. In the case of GMFT and CL, each short text *d*_*i*_ is represented as a binary vector ${\vec{x}}_{i}=\left\{b_{1},b_{2},\ldots ,b_{Q}\right\}$ indicating whether the corresponding term is included or not in the short text. In the case of BoW the texts are represented through real value vectors ${\vec{x}}_{i}=\left\{f_{1},f_{2},\ldots ,f_{Q}\right\}$, indicating the TF-IDF value (Aizawa, 2003) of each term in the short text.

In the case of the GMFT feature set, the 100 most frequent tokens in each sentiment category were used for the creation of the index of terms. This resulted in an index with a length equal to *Q* = 203 for the Facebook comments and *Q* = 178 for the tweets. In both cases, there were common tokens among the various categories, including, of course, Stopwords (n.d.). In the case of BoW, the indices for both Facebook comments and tweets are quite lengthy (3,166 and 3,151 respectively), and this length increases logarithmically with the number of samples (short texts) in the training set.

As already explained earlier, in the current study crowd intelligence regarding the short text classification was collected in the form of crowdtagging. Since every short text message was assessed by at least three annotators, we adopted two different crowd lexicon creation strategies, named CL2V (*Crowd Lexicon 2 Votes*) and CLMV (*Crowd Lexicon Majority Voting*). In the first, we included in the index tokens (tags) suggested by at least two annotators while in CLMV a token was included in the index if it was suggested by the majority of the annotators. We should note here, as can also be seen in Figure 1, that the annotators were instructed to select the tags from the body of short text (tweet and Facebook comments), thus crowd intelligence was collected in the form of token filtering and implicit sentiment understanding.

By comparing the length of CL2V and CLMV indices in tweets and Facebook comments, we see some interesting variations. Despite the fact that the number of Facebook comments used the training set (2,103) was almost twice the corresponding number of tweets (1,178), the length of the CL2V index for the Facebook comments (427) was significantly smaller than that of the tweets (746). A similar observation can be made for the length of CLMV indices. In addition, it appears, by comparing the lengths of CL2V and CLMV in each one of the two short text types, that the annotators tend to more easily agree on the important keywords in a tweet than in a Facebook comment. The overall conclusion we can draw based on these observations is that sentiment-related keywords can be more easily identified in tweets rather than in Facebook comments. This can be attributed to the presence of hashtags and mentions, but it also indicates that although both tweets and Facebook comments are short texts, they are not so similar as it appears in some studies (Barbosa and Feng, 2010).

Word Embeddings is an umbrella term for a set of language modeling approaches and feature extraction techniques in natural language processing (NLP), where tokens (words), phrases or short documents are mapped to vectors of real numbers. The typical size of these vectors is 100 but in fact there is no rule that can help you decide on the optimal vector length. Although pre-trained models, usually learned using the Wikipedia documents, do exist for practically each word (or more correctly token) that appears in the Web, this is obviously not the case for short texts and phrases. In that case, short text embeddings must be learned from scratch as explained by Le and Mikolov (2014). We have experimented extensively regarding the optimal vector length of word embeddings, for both *Doc2Vec* and *fasttext* methods, and we concluded that the typical vector size of 100 elements shows a slightly better performance independently of the classifier that is used.

### 3.4. Learning

In order to assess the appropriateness of each feature set on short text classification, both tweets and Facebook comments were first randomly separated into a training and a test set with an approximate ratio of 65:35. The corresponding distributions per category in these two sets are shown in Tables 2, 3. In both cases, one of the sentiment categories, that is the ‘Hate' category in tweets and the “Comparative” category in Facebook comments, is underrepresented. Thus, constructing good classification models becomes harder. In order to emulate as much as possible real operation situations, the index sets were developed from tokens found in the training sets. This is one of the reasons that the typical *k*-fold evaluation scheme was not adopted in our experiments. Short texts were represented as binary vectors (CMFT, CL2V, CLMV cases), as TF-IDF vectors (BoW case) or as real value vectors (*Doc2Vec* and *fasttext* case) in the related vector space produced by each one of the indices, as described in section 3.3.

**Figure 2:**
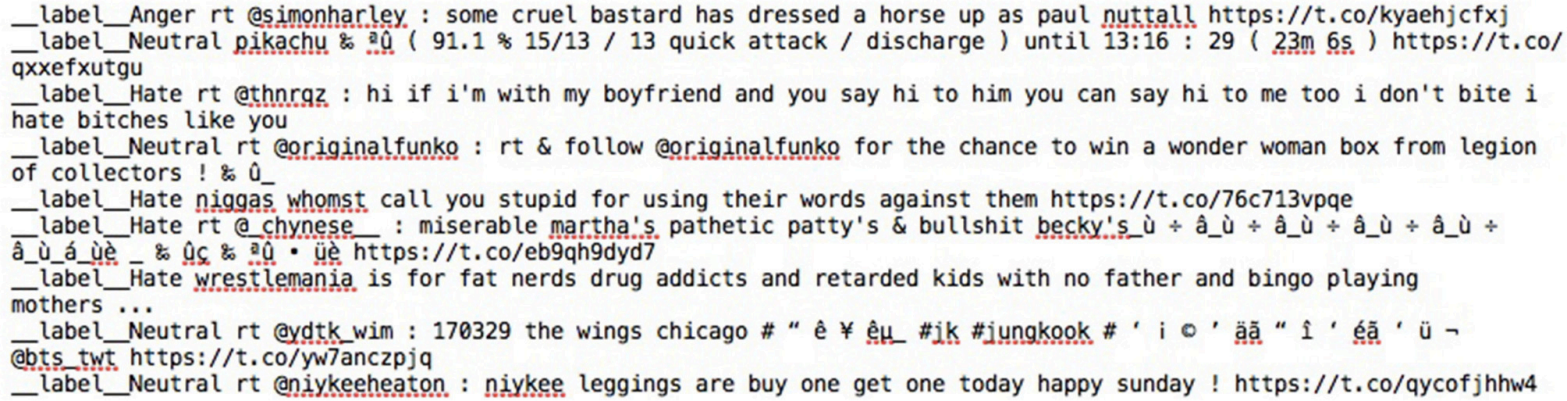
A screenshot of the file used for the training of the *fasttext* classifier.

Four different classifier types were constructed for each one of the previously-mentioned indices using the training sets. The case of *fasttext* was different since feature extraction and classifier learning are combined. The training in this case was done with the help of text files similar to the one shown in Figure 2. As usual the test sets, that is the unseen instances, were utilized for testing the effectiveness of the created classification models. A total of 21 classification models were constructed, for each one of the short text types, corresponding to five different vector spaces and four different learning algorithms along with the *fasttext* case (see Tables 4, 5). We used the learning algorithms' implementations of the Python module *Scikit-learn* (Pedregosa et al., 2011) as well as the corresponding Python library for fastText (2018). In order to have a fair comparison of the feature sets, all learning algorithms were used without tuning based on their default settings. Tokenization of both tweets and Facebook comments was achieved through the TweetTokenizer (n.d.) of the NLTK (n.d.) library. Note, however, that the *fasttext* classifier does not require any type of tokenization of the short text (see a snapshot of the training file in Figure 2) since it makes use of deep learning and the extracted features are based on character *n*-grams (Joulin et al., 2017).

**Table 4:** Comparison of feature sets and classifiers for sentiment classification of facebook comments.

**Feature set**	**Classifier**				**Average**
	**Decision trees**	**Linear SVC**	**Naive Bayes**	**Stoch. gradient descent**	
GMFT [203]	0.5928	0.7001	0.7353	0.6711	0.6748
CL2V [427]	0.6500	0.7142	**0.7432**	0.7071	**0.7036**
CLMV [202]	0.5858	0.6790	0.7361	0.6719	0.6682
BoW [3166]	**0.6640**	**0.7282**	0.6429	**0.7573**	0.6981
Doc2Vec [100]	0.4785	0.6289	0.6429	0.6438	0.5985
Deep learning [100]	**Fasttext**				**0.7282**

**Table 5:** Comparison of feature sets and classifiers for sentiment classification of tweets.

**Feature set**	**Classifier**				**Average**
	**Decision trees**	**Linear SVC**	**Naive Bayes**	**Stoch. gradient descent**	
GMFT [178]	0.6955	0.7072	**0.7511**	0.7233	0.7193
CL2V [746]	0.7628	0.7189	0.7350	0.6852	0.7255
CLMV [536]	**0.7687**	**0.7306**	0.7452	0.7086	**0.7376**
Bag of Words (3151)	0.7072	0.7291	0.7013	**0.7291**	0.7167
Doc2Vec [100]	0.5256	0.6750	0.6735	0.7013	0.6439
Deep learning [100]	**Fasttext**				**0.7291**

## 4. Experimental Results and Discussion

The classification performance, i.e., label prediction accuracy in regards to the gold standard, of the 21 compared classification models for the Facebook comments are summarized in Table 4 while Table 5 shows the corresponding results for the tweets' case. The reported values are average scores on 10 runs. As expected in the Naive Bayes case, the obtained performance does not change during the different runs since it does not include any randomly selected parameters. However, it turned out that the same happens in the case of the *fasttext* classifier. Since we do no have further information regarding the implementation of this classifier, we only assume that it is probably based on a rule-based approach without any random parameters or random initialization. We have avoided the typical *k*-fold evaluation because the emphasis of our work was on the feature sets' assessment rather than on the classifier performance. So, in order to avoid a further addition of randomness we kept our training and test sets fixed.

The analysis of results, presented herein, follows three axes. First we try to answer the main research question of this study, that is whether crowd intelligence, as expressed through crowdtagging, can create a more effective feature set than those extracted through deep learning as in *fasttext* and *Doc2Vec*. A comparison of sentiment analysis of tweets and Facebook comments follows, while a better way to collect crowd intelligence in the form of crowd-tagging is also discussed.

### 4.1. Statistical Analysis of the Results

Since the performance scores shown in Tables 4, 5 appear very close to each other, we ran a multi-way ANOVA test in order to identify the impact of each one of the factors, namely data source (tweets, facebook comments), feature type (GMFT, etc.) and classifier (Decision Trees, etc.). The results of the ANOVA test are summarized in Table 6. While the impacts of all three factors are discussed further—in a qualitative fashion—in sections 4.2 and 4.3, respectively, we can see in Table 6 an overview of the significance of their influence. According to the obtained *p*-values, the type of features affects significantly the retrieved accuracy scores since the probability to get, with a random feature set, an *F*-ratio higher than the computed one (i.e., 15.782) is *p* = 0.0001. Thus, we conclude that the feature sets that we have included in our study are indeed informative. A similar interpretation holds for the data source (tweets or facebook comments) and the classifier. We clearly see that the performance scores differ depending on the data source, which in simple words means that no similar performance should be expected for the categorization of tweets and Facebook comments. We should mention here, however, that the categories used and the data distribution per category differ in the tweets' and Facebook comments' case (see Tables 2, 3). Thus, the dependency of performance scores on the data source type is more or less expected.

**Table 6:** The results of the multivariate ANOVA test on the combined results of Tables 4, 5.

	**∑*x*^2^**	**df**	**F-ratio**	**p-value**
Data source	0.0160	1	21.618	0.00056
Feature set	0.0469	4	15.782	0.00010
Classifier	0.0284	3	12.760	0.00483
Data⊛feature	0.0035	4	1.173	0.37088
Data⊛classifier	0.0111	3	4.986	0.01792
Feature⊛classifier	0.0333	12	3.734	0.01528
Residual	0.0089	12		

It also appears that the classifier affects the obtained scores, which means that proper classifiers are needed for the tasks classifying tweets and Facebook comments. However, among the three influencing factors, the least important is the classifier. Thus, the problem itself, for example the type of data, the selected features, the number and kind of categories used and the data distribution per category, are more important than the selection of the classifier. This is further justified through the multiple comparison of means of the Tukey HSD test, which is reported in **Table 9**.

As far as the pairwise co-influences are concerned, we observed that co-influence of data source and feature set is insignificant while the co-influences of data source and classifier and feature and classifier, respectively, are approximately equally significant. The fact that the co-influence of data source and feature set is insignificant means that there is no evidence that there are feature sets, among the compared ones, that fit better with one or the other data source type. On the other hand, it turns out that there is a rather strong correlation among data source and the selected classifier, which means that there are combinations of classifiers and data sources that are more suitable than others. Similarly, as expected and further discussed in section 4.2, the combinations of feature sets and classifiers are important and, therefore, we conclude that there are feature types which are better suited for specific classifiers.

In an effort to make justified conclusions regarding the better combination of the three influencing factors, i.e., data source, feature set and classifier, we ran the pairwise Tukey Honest Significant Difference (HSD) *post hoc* test in each one of these three cases. The results of the Tukey HSD test are summarized in Tables 7–9. We first observed that better results are achieved in the tweet classification problem and, as we see in Table 8 the difference is significant and the null hypothesis that the performance scores on both tweets and Facebook comments are identical is rejected (see the last column in Table 8). For better interpretation of the results in Tables 7–9, we should mention here that the differences reported in columns 3 − 5 refer to the *G*_2_ − *G*_1_.

**Table 7:** The influence of feature set through multiple comparison of means - Tukey HSD, FWER=0.05.

***G*_1_**	***G*_2_**	**Meandiff**	**Lower**	**Upper**	**Reject**
BOW	D2V	−0.0862	−0.1635	−0.0089	True
BOW	GL2V	0.0072	−0.0701	0.0845	False
BOW	GLMV	−0.0041	−0.0814	0.0731	False
BOW	GMFT	−0.0103	−0.0876	0.067	False
D2V	GL2V	0.0934	0.0161	0.1707	True
D2V	GLMV	0.0821	0.0048	0.1593	True
D2V	GMFT	0.0759	−0.0014	0.1532	False
GL2V	GLMV	−0.0113	−0.0886	0.066	False
GL2V	GMFT	−0.0175	−0.0948	0.0598	False
GLMV	GMFT	−0.0062	−0.0835	0.0711	False

**Table 8:** The influence of data source through multiple comparison of means - Tukey HSD, FWER=0.05.

***G*_1_**	***G*_2_**	**Meandiff**	**Lower**	**Upper**	**Reject**
FC	TW	0.0401	0.0023	0.0778	True

**Table 9:** The influence of classifier through multiple comparison of means - Tukey HSD, FWER=0.05.

***G*_1_**	***G*_2_**	**Meandiff**	**Lower**	**Upper**	**Reject**
DT	LSVC	0.0580	−0.0114	0.1275	False
DT	NB	0.0676	−0.0019	0.1370	False
DT	SGD	0.0568	−0.0127	0.1262	False
LSVC	NB	0.0095	−0.0599	0.0790	False
LSVC	SGD	−0.0012	−0.0707	0.0682	False
NB	SGD	−0.0108	−0.0802	0.0587	False

The most obvious conclusion we draw from Table 7 is that the only feature set that is lacking behind is the *Doc2Vec* (noted as D2V in Table 7). We note here that these types of features are numerical (non-semantic) ones and are computed as combinations of word embeddings. The features of the *fasttext* approach are based on the same principle, but in this case they are combined with a classifier taking advantage of a deep level architecture. Regarding the rest of the feature sets, we do not observe a significant difference among them, although with closer investigation and by doing the necessary combinations the best results are obtained with the GL2V feature set. As far as the classifiers are concerned, we see in Table 9 that no significant differences are detected. The Decision Trees seem to have a slightly worse performance than the other three classifiers, with the Naive Bayes being marginally better than the others.

### 4.2. Crowd Intelligence vs. Deep Learning

We see in the sentiment classification of tweets (Table 5) that a combination of the *crowd lexicon* with a Decision Tree classifier clearly outperforms, by 4%, the *fasttext* classifier that is based on features extracted through deep learning. This outcome is also qualitatively supported via a close inspection and comparison of Tables 10, 11. The recall value of the smaller of the three categories, i.e., the “Hate” category, is significantly higher in the *crowd lexicon* (Table 10) than that of deep learning (Table 11). This result shows that the sentiment keywords proposed by the crowd help to create effective classification models even in cases where the dataset is unbalanced in terms of the samples' distribution per category. The fact that in tweets the *crowd lexicon* and Decision Tree combination achieves the best performance is also in full agreement with the findings of Tsapatsoulis and Djouvas (2017).

**Table 10:** Confusion matrix for the tweets in the best combination, i.e., the CLMV feature set - *Decision Trees* classifier.

**Category**	**Anger**	**Hate**	**Neutral**	**Recall**
Anger	**174**	26	57	0.6770
Hate	34	**79**	23	0.5809
Neutral	17	1	**272**	0.9379
Precision	0.7733	0.7453	0.7727	**0.7687**

**Table 11:** Confusion matrix for the *fasttext* classifier for the tweets.

**Category**	**Anger**	**Hate**	**Neutral**	**Recall**
Anger	**178**	15	64	0.6926
Hate	45	**64**	27	0.4706
Neutral	30	4	**256**	0.8828
Precision	0.7036	0.7711	0.7378	**0.7291**

The case of sentiment classification of Facebook comments (see Table 4) is more complicated. First, the best performance is achieved with the classic bag-of-words representation in combination with the Stochastic Gradient Descent learning algorithm (BoW-SGD), while a combination of *crowd lexicon* with Naive Bayes follows. Thus, we can conclude that the lengthy indices of terms lead to better classification performance. This, in turn, shows that keyword-based indices lead to effective sentiment classification models for Facebook comments, but the quality of the selected keywords is of primary importance. In the context of crowdtagging systems this can be facilitated by increasing the number of assessments per Facebook comment, combined with an intelligent tag selection scheme as suggested by Giannoulakis et al. (2017).

In Tables 12, 13, we see the confusion matrices of sentiment classification of Facebook comments of the BoW-SGD combination and *fasttext*. The low recall values of the smaller category, i.e., the “Comparative” category, show an ineffective modeling case. The performance of the BoW-SGD combination, though, is much better than that of *fasttext*. Thus, better selection of keywords related to the “Comparative” category could help on the overall improvement of sentiment classification of Facebook comments using indices of terms. On the contrary, this is very unlikely to happen with the case of features extracted through deep learning.

**Table 12:** Confusion matrix for the facebook comments in the best combination, i.e., BoW feature set - *Stochastic Gradient Descent* classifier.

**Category**	**Comparative**	**Negative**	**Neutral**	**Positive**	**Recall**
Comparative	**56**	28	27	18	0.4341
Negative	10	**252**	11	20	0.8601
Neutral	13	35	**194**	65	0.5668
Positive	9	17	23	**359**	0.8799
Precision	0.6364	0.7590	0.7608	0.7771	**0.7573**

**Table 13:** Confusion matrix for the *fasttext* classifier for the facebook comments.

**Category**	**Comparative**	**Negative**	**Neutral**	**Positive**	**Recall**
Comparative	29	**54**	37	9	0.2248
Negative	10	**233**	11	39	0.7952
Neutral	1	27	**207**	72	0.6743
Positive	1	7	41	**359**	0.8799
Precision	0.7073	0.7259	0.6993	0.7495	**0.7282**

The Word Embeddings representation itself, that is the case of *Doc2Vec*, leads to disappointing performance across all classifiers in the case of Facebook comments, while in tweets a decent performance is achieved only with the stochastic gradient descent learning algorithm. If we contrast this performance with the one achieved by the *fastext*, we conclude that word embeddings do not fit well with typical machine learning algorithms, but they require a specially designed classifier. Unfortunately, details regarding the classifier type used in *fasttext* are not publicly available.

### 4.3. Tweets vs. Facebook Comments and the Role of Classifier Type

It has been already mentioned in section 3.3 that it is more difficult to extract keywords from Facebook comments than from tweets, probably due to the wide use of hashatgs, mentions and emoticons in the latter. This difficulty is reflected on the performance of the Decision Tree classifier as it can be observed in Tables 4, 5. In the case of tweets, the Decision Trees show excellent performance, even when combined with low- to medium-sized indices of terms as in the cases of CMFT, CL2V, and CLMV. On the contrary, for the Facebook comments a large index is required, i.e., the BoW case, to develop a fairly performing Decision Tree classifier. Thus, selection of appropriate keywords has a high impact on the quality of Decision Tree classifier that is learned. We emphasize Decision Trees here because, among all compared machine learning algorithms, this is the one that better fits human logic due to its rule-based nature.

Among the other classification models, it appears that those based on Naive Bayes are less affected by the differences between tweets and Facebook comments. The *fasttext* classifier shows also a stable performance in both short text types, but this is expected since the type of features it uses are basically character *n*-gram combinations and not tokens.

### 4.4. How to Collect Crowd Intelligence in Crowd-Tagging Systems

Tag selection in crowd-tagging systems is typically accomplished through a voting scheme. This scheme makes use of simple rules, such as full agreement among annotators, majority voting or agreement of at least two annotators, or sophisticated weighting schemes, such as the HITS algorithm as suggested by Giannoulakis et al. (2017). As already explained before, the CL2V and CLMV indices, used in the current work, are based on the two annotator agreement rule and on the majority voting rule respectively. The full agreement rule is rarely used in crowd-tagging systems with more than three assessments per object (herein short texts), whereas, the HITS algorithm is beneficial for situations where many assessments (typical more than 10) per object are available.

We see in Table 5 that tweet classification models that are based on the CLMV index show slightly better performance than those constructed using the CL2V index. The difference, however, is not statistically significant. The situation is totally reversed in the case of sentiment classification of Facebook comments, as can be seen in Table 4. In this case, the models that are based on the CL2V index clearly outperform those constructed using the CLMV index. The difference in performance is quite large independently of the learning algorithm used, with the exception of Naive Bayes classifier where the difference is insignificant. Taking into account that the best-performing classification models for Facebook comments are based on the bag-of-words (BoW) method, which makes use of very large indices of terms, we can conclude that a more relaxed tag selection process is beneficial for crowd-tagging systems that are aiming for Facebook comment classification schemes.

## 5. Conclusion and Further Work

The main research question of the current study was to compare the effectiveness of features indicated by humans (i.e., keywords) with those extracted through deep learning in regard to sentiment classification of short texts and tweets and facebook comments in particular. We have empirically shown that the human-created indices, called *crowd lexicon* herein, that are based on crowdtagging, can be effectively used for training sentiment classification models for short texts and that those models are at least as effective as the ones that are developed through deep learning or even better. This result is in line with the findings of our previous study (Tsapatsoulis and Djouvas, 2017) in which we showed that the tokens (unigrams), indicated by humans, lead to classification models with the highest performance regarding tweet classification. The models that use this feature set, consistently and independently of the machine learning algorithm adopted, surpass any other model in terms of tweet classification performance.

We have also demonstrated that the peculiarities of tweets classification compared to Facebook comment classification, regarding the feature selection process, are not so small. Identifying sentiment-related keywords in Facebook comments is more difficult than in tweets. The presence of hashtags and mentions probably helps keyword selection in tweets. This, in turn, has a significant impact on the best-performing classification model that can be developed. Good keywords lead to effective Decision Tree classifiers, which in the case of sentiment classification of tweets outperform any other classification model. On the contrary, classification models based on Decision Trees show poor performance in the case of sentiment classification of Facebook comments. As an intermediated case, the deep learning classifiers (i.e., *fasttext*), which are basically combined character *n*-grams, perform similarly on tweets and Facebook comments.

The way collective knowledge is gathered in crowd-tagging systems is another important issue. In contemporary crowdsourcing platforms the number of annotators can be as large as we want. This allows for different token selection strategies to form the human-created indices (*crowd lexicon*). A token can be added to the crowd lexicon in case it is suggested by all annotators, the majority of annotators, at least two annotators or through a more sophisticated approach such as the HITS algorithm (Giannoulakis et al., 2017). The cases of majority voting and two annotator agreement were investigated in this study. The full agreement case leads to very short indices of terms, especially whenever many annotators are involved in the crowdtagging process. Majority voting and two annotator agreement show similar performance in the case of tweets, whereas the two annotator agreement approach leads clearly to more effective indices of terms in the case of Facebook comments, showing once again that the process of identifying sentiment keywords is much more difficult in Facebook comments than in tweets.

We did not investigate, in the current study, feature set combination since our primary aim was to compare crowd intelligence with deep learning-related features and not to develop the best classifier for sentiment classification of short texts. Some studies (Hamdan et al., 2015) claim improvement on the classification performance through feature sets' combination via properly selected weighting schemes. A combination of classification models through voting schemes is also another alternative that deserves further investigation. We will experiment with these issues in future research.

As indicated in previous studies (Hamdan et al., 2015; Shirbhate and Deshmukh, 2016), the existence of hashtags, emoticons and slang words in tweets favors the unigram method. Is this conclusion valid for other short text types such as Facebook comments? Or are bigram (or generally *n*-gram tokens)-based features that cope also with negation (Pak and Paroubek, 2010) more effective? We are currently working on these research questions using the same approach as the one followed by Tsapatsoulis and Djouvas (2017).

## Author Contributions

All authors listed have made a substantial, direct and intellectual contribution to the work, and approved it for publication.

### Conflict of Interest Statement

The authors declare that the research was conducted in the absence of any commercial or financial relationships that could be construed as a potential conflict of interest.
